# Fetal development of functional thalamocortical and cortico–cortical connectivity

**DOI:** 10.1093/cercor/bhac446

**Published:** 2022-12-15

**Authors:** Athena Taymourtash, Ernst Schwartz, Karl-Heinz Nenning, Daniel Sobotka, Roxane Licandro, Sarah Glatter, Mariana Cardoso Diogo, Polina Golland, Ellen Grant, Daniela Prayer, Gregor Kasprian, Georg Langs

**Affiliations:** Computational Imaging Research Lab, Department of Biomedical Imaging and Image-guided Therapy, Medical University of Vienna, Waehringer Guertel 18-20, A-1090 Vienna, Austria; Computational Imaging Research Lab, Department of Biomedical Imaging and Image-guided Therapy, Medical University of Vienna, Waehringer Guertel 18-20, A-1090 Vienna, Austria; Computational Imaging Research Lab, Department of Biomedical Imaging and Image-guided Therapy, Medical University of Vienna, Waehringer Guertel 18-20, A-1090 Vienna, Austria; Center for Biomedical Imaging and Neuromodulation, Nathan Kline Institute, 140, Old Orangeburg Road, Orangeburg, NY 10962, United States; Computational Imaging Research Lab, Department of Biomedical Imaging and Image-guided Therapy, Medical University of Vienna, Waehringer Guertel 18-20, A-1090 Vienna, Austria; Computational Imaging Research Lab, Department of Biomedical Imaging and Image-guided Therapy, Medical University of Vienna, Waehringer Guertel 18-20, A-1090 Vienna, Austria; Laboratory for Computational Neuroimaging, A.A. Martinos Center for Biomedical Imaging, Massachusetts General Hospital and Harvard Medical School, Bldg. 149, 13th Street, Charlestown, MA 02129, United States; Division of Neuroradiology and Musculoskeletal Radiology, Department of Biomedical Imaging and Image-guided Therapy, Medical University of Vienna, Waehringer Guertel 18-20, A-1090 Vienna, Austria; Division of Neuroradiology and Musculoskeletal Radiology, Department of Biomedical Imaging and Image-guided Therapy, Medical University of Vienna, Waehringer Guertel 18-20, A-1090 Vienna, Austria; Radiology Department, Hospital CUF Tejo, Av. 24 de Julho 171A, 1350-352 Lisboa, Portugal; Computer Science and Artificial Intelligence Laboratory, Massachusetts Institute of Technology, 77, Massachusetts Avenue, Cambridge, MA 02139, United States; Fetal-Neonatal Neuroimaging and Developmental Science Center, Boston Children's Hospital, Harvard Medical School, 300, Longwood Avenue, Boston, MA 02115, United States; Division of Neuroradiology and Musculoskeletal Radiology, Department of Biomedical Imaging and Image-guided Therapy, Medical University of Vienna, Waehringer Guertel 18-20, A-1090 Vienna, Austria; Division of Neuroradiology and Musculoskeletal Radiology, Department of Biomedical Imaging and Image-guided Therapy, Medical University of Vienna, Waehringer Guertel 18-20, A-1090 Vienna, Austria; Computational Imaging Research Lab, Department of Biomedical Imaging and Image-guided Therapy, Medical University of Vienna, Waehringer Guertel 18-20, A-1090 Vienna, Austria; Computer Science and Artificial Intelligence Laboratory, Massachusetts Institute of Technology, 77, Massachusetts Avenue, Cambridge, MA 02139, United States

**Keywords:** fetal brain development, functional connectivity, magnetic resonance imaging, thalamus

## Abstract

Measuring and understanding functional fetal brain development in utero is critical for the study of the developmental foundations of our cognitive abilities, possible early detection of disorders, and their prevention. Thalamocortical connections are an intricate component of shaping the cortical layout, but so far, only ex-vivo studies provide evidence of how axons enter the sub-plate and cortex during this highly dynamic phase. Evidence for normal in-utero development of the functional thalamocortical connectome in humans is missing. Here, we modeled fetal functional thalamocortical connectome development using in-utero functional magnetic resonance imaging in fetuses observed from 19th to 40th weeks of gestation (GW). We observed a peak increase of thalamocortical functional connectivity strength between 29th and 31st GW, right before axons establish synapses in the cortex. The cortico–cortical connectivity increases in a similar time window, and exhibits significant functional laterality in temporal-superior, -medial, and -inferior areas. Homologous regions exhibit overall similar mirrored connectivity profiles, but this similarity decreases during gestation giving way to a more diverse cortical interconnectedness. Our results complement the understanding of structural development of the human connectome and may serve as the basis for the investigation of disease and deviations from a normal developmental trajectory of connectivity development.

## Introduction

Functional specialization of the mammalian brain depends to a large extent on thalamocortical patterning signals (Sherman and Guillery 2013). As a central hub, the thalamus relays nearly all incoming and outgoing information to and from the cortex and mediates cortico–cortical communication (Jones 2001). From ex-vivo studies, we understand the structural substrate of emerging thalamocortical connectome. Early thalamocortical afferent outgrowth occurs as early as 8–10 post-conceptual week (PCW) at the level of the *thalamic Anlage* (Kostović and Judaš 2006, 2010). From 19 to 22 PCW (21 and 24 weeks of gestation), thalamocortical afferents accumulate in the superficial subplate, known as a “waiting” compartment for the afferent fibers (Kostovic and Rakic, 1984a, 1990; Rakic et al. 1977). Between 24 and 26 PCW, thalamocortical afferents invade the cortical plate in a deep-to-superficial fashion. With the development of primary gyri and sulci between 29 and 32 PCW, thalamocortical axons establish synapses with cortical plate layer IV neurons and form the earliest cortical circuitry. As a result, sensory-driven cortical activations by somatosensory, auditory, and visual stimuli are expected at this time (Kostović and Judaš 2010; Vanhatalo and Kaila 2006). The period between 33 PCW and term is then dominated by interhemispheric synchronization, gradual resolution of the subplate, and maturation of long intrahemispheric (associative) and interhemispheric (callosal) cortico–cortical connectivity (Kostović and Judaš 2006; Krsnik et al. 2017). As the observation of function in utero is challenging, until now, we lack a quantitative model of the development of the associated thalamocortical functional connectome and how it is shaping the accompanying global cortico–cortical connectivity architecture before birth.

Over the last decades, animal experiments and postmortem studies on human fetuses have provided useful insights into the early formation of thalamocortical synapses. However, their functional growth and interaction with cortical circuit formation in living human fetuses have been much less studied. With the recent advances in imaging techniques, structural and functional organization of thalamocortical connections of preterm and term infants was examined in several studies. Notably, these previously published studies revealed that thalamus–sensorimotor and thalamus–salience networks are already present at term, while thalamus–medial visual and thalamus–default mode networks are not evident until 1 year of age (Alcauter et al. 2014; Cai et al. 2017). Furthermore, extreme prematurity was found to be associated with decreased connectivity between the thalamus and prefrontal, insular, and anterior cingulate cortex as well as increased connectivity with the primary sensory cortex (Ball et al. 2013; Toulmin et al. 2015). Thalamocortical structural connectivity patterns in the preterm brain measured at term equivalent age were correlated with cognitive performance in early childhood (Ball et al. 2015; Jakab et al. 2020; Toulmin et al. 2021). Thalamic segmentation based on the dominant structural and/or functional connections with cortical networks revealed adult-like topology with a more widespread representation of associative cortex than primary cortex on the thalamus (Toulmin et al. 2015) and greater agreement between two modalities for primary-sensory cortices compared with higher order association areas such as temporal and posterior parietal cortices (Ferradal et al. 2019).

Despite the insights into network formation and function provided by these studies, the resulting patterns may not describe normal brain development before birth. As prematurity affects brain development on every hierarchical level (Vasung et al. 2019), the underlying reasons for preterm delivery or environmental factors may not be segregated from neurological processes in these cases. Moreover, exposure to the extrauterine environment and deprivation from key intrauterine signals in late pregnancy related to preterm birth seems to shape the infant brain differently than exposure to normal, full-term pregnancy (van den Heuvel and Thomason 2016). Therefore, in vivo studies on normally developing fetuses are needed to better understand the link between the development of brain anatomy and functional connectivity. Reference models of normal functional fetal brain development may enable explaining neurodevelopmental disorders or adverse outcomes such as a deviation in shape, variability, or timing.

In-vivo examination of structural brain asymmetry in the fetal period using in utero magnetic resonance imaging (MRI) has been reported in several studies, observing for instance the relatively earlier emergence of sulci and gyri of the right hemisphere in the perisylvian region (Kasprian et al. 2011; Habas et al. 2012). However, studies on prenatal hemispheric differences in the functional and structural connectome are scarce. (Thomason et al. 2014) assessed the modularity of the brain in typically developing fetuses between 19 and 39 weeks gestational age using resting-state functional MRI (rs-fMRI) and found a ventral frontal-temporal cortex module appeared to become more left-lateralized in older fetuses which potentially develop into Broca’s and Wernicke’s Areas. Ex-vivo tractography of postmortem human fetuses revealed no hemispheric asymmetry of the thalamocortical pathways during development and a higher number and volume of the anterior thalamocortical pathways compared with posterior regions (Takahashi et al. 2012; Qiaowen et al.. 2016; Wilkinson et al. 2017). In addition, global cortical gene expression profiles seem to be generally symmetrical at fetal stage (Pletikos et al. 2014), suggesting a large effect of intrauterine and extrauterine environment on brain lateralization (Vasung et al. 2020).

Here, we characterized the development of functional thalamocortical connectivity in normal fetuses using in-utero rs-fMRI and compared the emergence of connectivity patterns with those found in postmortem studies using histochemical methods and preterm born infants without any detectable brain abnormalities. We examined the time point at which synchronized activity between the thalamus and cortical regions of interest (ROIs) occurs, and modeled its connectivity pattern during gestation. In addition, we investigated the relationship between the development of thalamocortical and cortico–cortical connectivity across the cortex and assessed functional interhemispheric asymmetry during the second and third trimesters. Based on previous studies, we hypothesized that functional thalamocortical connectivity increases during gestation and its pattern of development is similar in homologous regions of two hemispheres.

## Methods

**Fig. 1:**
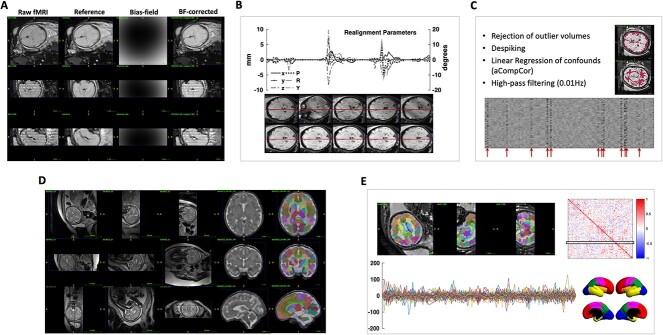
Single subject processing pipeline from raw fMRI data to subject-specific functional connectivity map. A) correction of signal nonuniformity; B) motion correction; C) rejection of outlier volumes, nuisance regression, and temporal filtering; D) super-resolution 3D reconstruction of the raw T2 images and the resulting segmentation; E) registration of the segmentation to the fMRI space, clean bold signal extracted from each segment (by averaging across voxels), resulting functional connectivity map, and illustration of the cortical lobes on a customized fetal surface.

Figure 1 illustrates the complete processing pipeline from raw fMRI data to subject-specific functional connectivity map. We addressed the technical challenges inherent to in-utero imaging such as excessive fetal movement, signal nonuniformity, and low SNR with established image processing methods described below.

### Participants and image acquisition

This study included 72 fetuses recruited from routine clinical examinations at Vienna General Hospital (AKH). The average gestational age of subjects was 28+0.6 (range: 19+5–39+2) weeks. None of the cases showed any neurological pathologies. Pregnant women were scanned on a 1.5T clinical scanner (Philips Medical Systems, Best, Netherlands) using a sensitivity encoding (SENSE) cardiac coil with five elements, and no contrast agents or sedatives were administered. Consecutive T2-weighted scans were acquired in approximate axial, coronal, and sagittal planes of the fetal brain with an in-plane resolution of 0.78–0.9 $mm$ and slice thickness of 3–4.4 $mm$. These scans were used to obtain individualized segmentations as will be explained in the next section. Moreover, blood-oxygenation level-dependent (BOLD) images were acquired with following the parameters: TR/TE = 3000/50 ms, matrix size of 144$\times $144, in-plane resolution = 1.74$\times $1.74 $mm^{2}$, slice thickness = 3$mm$, flip angle = 90$^{\circ }$, and 96 volumes per acquisition. During each TR interval, 18 slices were acquired with interleaved slice ordering (1-4-7...2-5-8...3-6-9..) to minimize cross-talk between adjacent slices.

The study protocol was approved by the institutional ethical boards at AKH and Medical University of Vienna, and research was conducted according to the principles expressed in the Declaration of Helsinki.

### Thalamus and cortical ROI definition

For all subjects, a combined registration and slice-wise motion correction of T2 weighted images followed by a robust super-resolution technique were applied to reconstruct an isometric volume with a resolution of $1mm^{3}$. Automatic atlas-based segmentation was carried out using a publicly available atlas of fetal brain anatomy (Gholipour et al. 2017). As a result, 78 cortical ROIs and six subcortical structures including the thalamus were consistently found in all subjects (see Supplementary Material for the details of the structural data processing as well as the list of extracted cortical ROIs in Supplementary Fig. S4 (Schwartz et al. 2016, 2021).

### rs-fMRI preprocessing

Signal nonuniformity caused by spatially variable sensitivity of the receiver coil was corrected as the first step of preprocessing for all BOLD images. We adopted the N4ITK algorithm according to the proposed approach (Turk et al. 2017) for in vivo fetal fMRI in which a reference volume was first created by averaging less contaminated volumes in the entire time series and then a single 3D bias field was estimated from the manual delineated brain mask on the reference volume. Each volume was subsequently divided by the same 3D bias field estimate. The second part of the pipeline consists of both inter-slice and inter-volume motion correction using the technique initially suggested by (You et al. 2016). For this, the original 4D BOLD sequence of length L with time interval $\triangle $t (=TR) was temporally expanded into a sequence of length 3L with interval $\triangle $t/3 by alternating between every third slice as it was acquired with interleaved scan. The missing slices in each expanded volume were filled using shape-preserving piecewise cubic interpolation over slices. Subvolumes were then registered together and recombined via voxelwise averaging to generate volumes at the original resolution. For motion correction across different volumes, we employed a pairwise registration method between the reference volume as a fixed image and the rest of the volumes in the series as moving images. Next, the outlier volumes whose severe motion artifact could not be fully compensated by the motion correction algorithm were automatically detected and excluded from further analysis. This is achieved by identifying voxels where an unexpected change of intensity in the temporal domain occurs and then rejecting those volumes with more than %5 of outlier voxels. Supplementary Fig. S2 shows the percentage of volumes kept after volume outlier rejection. Simultaneous temporal filtering (0.008 Hz) and nuisance regression were subsequently performed as suggested in (Hallquist et al. 2013). Nuisance regressors included the top five principal components (PCs) from white matter (WM) and the top five from cerebrospinal fluid (CSF) extracted from the subject-specific anatomical masks, a method known as *aCompCor* (Behzadi et al. 2007). To avoid the potential introduction of edge effects due to temporal filtering, the rejected frames were replaced using b-spline interpolation. The interpolated frames were not considered in the correlation analysis. Given that the thalamus is a small structure, particularly in our cohort, to avoid signal blurring we did not perform any spatial smoothing.

### Functional connectivity analysis

Functional connectivity analysis was performed on subjects’ level in the native functional space. For this, individualized masks for thalamic and cortical ROIs were mapped to the fMRI space using a rigid transformation computed between each individual structural T2 scan and the first volume of the fMRI data. Then, subject-specific functional connectivity matrices were obtained by correlating the average time-course of the BOLD signals in thalamic and cortical ROIs using Pearson’s correlation coefficient. As ROIs were individually specified, and not the entire fetal cortex was covered in the fMRI acquisition of all cases, we used the 78 ROIs that were consistently detected in all subjects; as a result, each subject’s connectome was represented by a 78$\times $78 matrix.

### Quality control of fMRI data

Irregular and unpredictable fetal movement is the most common artifact observed in in-utero fMRI. As there is no ground truth for the brain’s functional structure, especially before birth, quantifying the quality of data and the resulting FC maps is challenging. Here, we have developed two automatic approaches for the subject- and group-level evaluation of the data quality. Subject-specific assessment is based on the fact that nonstationary sources of motion, like fetus movement, can potentially induce changes in FC over time. So we have measured the association between dynamic FC and the estimated framewise displacement using a sliding window approach as described in (Taymourtash et al. 2019). The underlying assumption for group-level analysis is that distance between FC matrices and distance between GWs is correlated in a homogeneous group of subjects. Using Riemannian geometry and an iterative approach, all FC matrices were projected to tangent space, and subjects with a negative correlation between their distance to all other’s GW and FC were removed.

### Modelng the development of functional connectivity

To characterize the longitudinal development of thalamocortical and cortico–cortical connections, linear and nonlinear (sigmoid function) models were built as follows:

(1) \begin{align*}& f_{i}(t)=\frac{\beta_{1}}{1+exp\left[- \frac{t_{i}-\beta_{3}}{\beta_{4}}\right]}+\beta_{2}, \end{align*}

where $f_{i}$ describes functional connectivity of a fetus $i$ governed by the gestational age (time) variable $t_{i}$. This model was fit to each thalamic and cortical ROIs independently. $\beta _{2}$ and $\beta _{1}$ show the initial and final plateau of the model and $\beta _{4}$ describes its growth rate. The time instant $\beta _{3}$ indicates the inflection point of the sigmoid, where the first derivative $df/dt$ attains its peak and hence corresponds to the maximum increase of the connectivity. The robustness of the estimation of each model’s parameters was evaluated by a bootstrapping procedure over 1000 iterations. In addition, the adjusted R-squared values as a measure of goodness of fit were assessed by permutation testing to determine whether or not these models can significantly explain the developmental trajectories of thalamo/cortico–cortical connectivity with age. In all analyses, the significance level was set to 0.05 after correcting for multiple comparisons.

Furthermore, we evaluated subject-specific functional similarity and laterality for each cortical ROI and showed their changes during second and third trimesters. Similarity matrix was constructed by measuring the correlation coefficient between the connectivity profile of cortical ROI and laterality as implemented here relies on the basic computation LI = (Left - Right) / (Left + Right), where Left and Right represent nodal degrees after thresholding negative and weak connections. Therefore, a negative value of LI indicates a right hemispheric dominance and a positive value indicates a left-hemispheric dominance.

## Results

Resting-state fMRI was obtained from routine clinical examination of 72 singleton fetuses between 19+5 and 39+2 weeks of gestation (week+day). Fetal-specific image reconstruction and analysis including automated tissue labeling, segmentation, and artifact removal as illustrated in Fig. 1 and described in detail in the “Methods” section were used to process the fetal rs-fMRI data. 78 cortical parcels were consistently found by an automatic atlas-based segmentation of each subject’s T2-weighted image using a publicly available atlas of fetal brain anatomy (Gholipour et al. 2017). Subject-specific functional connectivity maps were then obtained by correlating the average time series of the BOLD signals in cortical and thalamic parcels. From the initial cohort, we excluded 13 cases due to excessive fetal movement, 10 cases due to temporal inconsistency of the achieved FC maps with gestational age, and 1 case with incomplete acquisition of BOLD scans. The final study sample consisted of 48 fetuses that had no confirmed brain abnormalities (mean gestational age $\pm $SD: 28.97$\pm $4.84 weeks). The exclusion of subjects was performed automatically during pre-processing of data based on the number of rejected volumes and the developed quality assurance and benchmarking tools discussed in the Supplementary Material. The average percentage of outlier volumes in the rs-fMRI time series for the remaining subjects was 6.14% (range:0–25%). It was marginally correlated with gestational age ($R=-0.26, P=0.048$) and significantly decreased after our method of motion correction compared with the original data ($P=0.032$). Fetal head movements quantified by either the mean or the maximum of the frame-wise displacement did not correlate with fetal age ($P=0.56$ and $0.26$, respectively) and so were not included in the regression analysis as confound variables.

### Development of functional thalamocortical connectivity

**Fig. 2:**
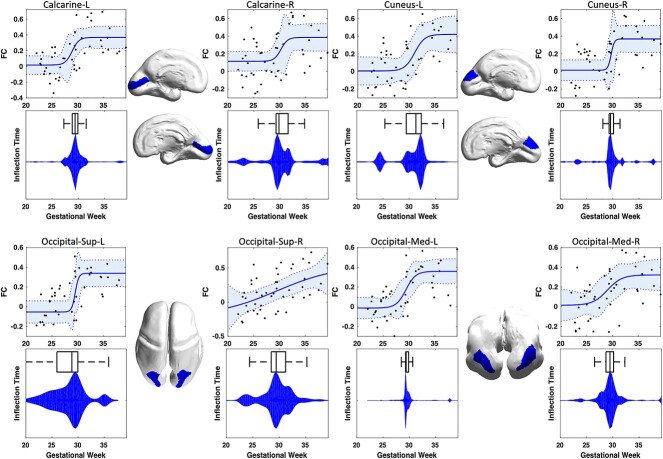
Association between the strength of thalamocortical FC and gestational age of fetuses. Each panel shows the estimated nonlinear growth trajectory, the distribution of the achieved inflection times, and the projection of each region onto a surface rendering of a 26-weeks-old fetal brain.

Functional thalamocortical connectivity increased significantly from 19 to 40 GW within the visual cortex including Calcarine (L/R), Cuneus (L/R), Superior-Occipital (L/R), and Medial-Occipital (L/R) regions tested at *P*$\leq $0.05 after correction for multiple comparisons (Fig. 2). The relationship between gestational age and functional connectivity was best described by a Sigmoid model (Table 1) with inflection points ranging from $28.79\pm 4.13$ for left-superior-occipital to $31.28\pm 2.88$ for left-cuneus. There were no significant differences between the estimated interval for inflection points and the time window of invasion and penetration of thalamocortical afferents into the cortical plate based on the Kostovic model (all *P*¡0.001). The distribution of the estimated inflection points for contralateral homologous parcels were found to be similar, and statistical tests for asymmetry of their developmental trajectories did not reach statistical significance (e.g. Calcarine L/R: t-stat=9.57, CI=0.88–1.34, *P*¡0.001). Developmental trajectories for other parcels in different cortical lobes were also computed, and can be found in the Supplementary Information section.

**Table 1:** Regional characteristics of prenatal intrinsic thalamocortical functional connectivity development: The accuracy of the model was evaluated in terms of goodness of fit criteria via bootstrapping over 1000 iterations. ns: not significant, na: not applicable, MOG: Middle Occipital Gyrus, SOG: Superior Occipital Gyrus; IOG: Inferior Occipital Gyrus, CAL: Calcarine fissure; CUN: Cuneus, LIN: Lingual gyrus, FFG: Fusiform gyrus.

	Sigmoid model		Model criterion				
Parcel	Initial plateau	Final plateau	Inflection time	Growth rate	Adjusted-R$^2$	AIC	BIC
MOG.L	-0.0133	0.3587	29.3857	0.4614	0.46	-24.85	-19.24
MOG.R	0.0149	0.3225	28.9932	0.3098	0.32	-14.16	-8.42
SOG.L	-0.0535	0.3389	29.5098	1.2673	0.47	-11.98	-6.51
SOG.R	-0.2091	0.5410	29.5015	0.0730	0.31	-2.35	3.63
CAL.L	0.3681	0.0173	28.8726	-0.6535	0.43	-15.03	-9.43
CAL.R	0.3858	0.1150	30.2688	-0.6667	0.24	-8.64	-2.65
CUN.L	0.0057	0.4203	31.0579	0.4492	0.39	-3.45	1.73
CUN.R	0.0144	0.3685	29.7047	1.4115	0.38	-5.57	-0.10
LING.L	0.0614	0.1593	26.9146	114.9882	0.07	-5.63	-0.17
LING.R	0.19	0.0496	24.9324	-28.9825	0.10	-10.45	-5.12
FFG.L	0.12	0.027	30.46	43.44	0.07	-24.32	-18.72
FFG.R	0.04	0.0804	24.2580	45.6220	0.02	-20.32	-15.28

### Development of functional cortico–cortical connectivity

**Fig. 3:**
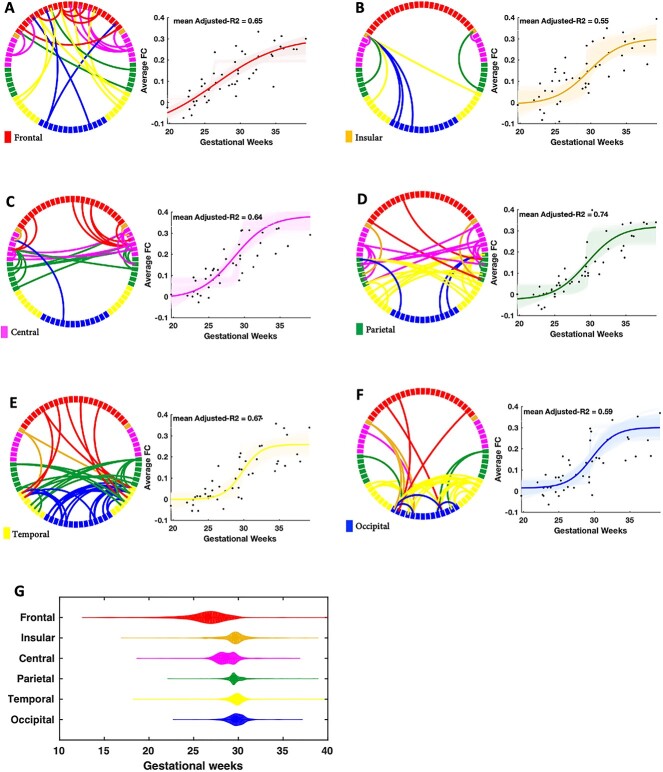
Development of cortico–cortical FC across gestational age of fetuses. Each circular graph depicts one cortical lobe with restricted edges whose development showed a significant correlation with gestational age after correcting for multiple comparisons. The violin plots show the distribution of the estimated inflection times for each cortical lobe.

Among 3003 distinct cortico–cortical functional connections (i.e. upper triangle of the 78$\times $78 brain connectivity matrix), we found 93 connections, between 58 distinct cortical parcels, whose development were significantly correlated with gestational age after correcting for multiple comparisons (*P*¡0.05, FDR corrected). These connections, separately for each cortical lobe, along with their development are illustrated in Fig. 3. Increased functional connectivity in older fetuses compared with younger ones was observed for all brain lobes with mean adjusted R$ ^2$ ranging from 0.55$\pm $0.08 for insular to 0.74$\pm $0.06 for the parietal lobe as determined by the bootstrapping procedure.

When comparing the inflection time of the sigmoid model for thalamocortical- and cortico–cortical connections, no significant difference was found in any of the cortical regions that were modeled by sigmoid function (all *P*>0.16). We did not find any significant difference between cortical lobes ($F(5,12)=1.4, P=0.34$) and between brain hemispheres ($F(1,12)=1.1, P=0.34$) in terms of the number of connections whose development were significantly related to gestational age. The overall estimated inflection point for each cortical lobe is illustrated in Fig. 3G. Our sample did not reveal a significant sequence of maturation in cortical lobes as the estimated distribution for the inflection time of different lobes didn’t differ significantly from each other (*P*=0.41).

### Similarity between mirrored connectivity profiles of homologous parcels decreases during gestation

**Fig. 4:**
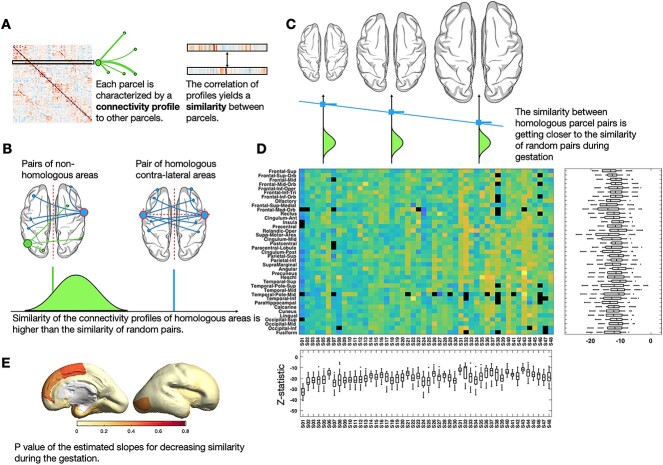
Similarity analysis. For a single subject, a similarity map was was quantified as Pearson’s correlation coefficient between their functional connectivity profiles (A) and was tested for homologous parcels (B). We observed the similarity between homologous parcels is significantly higher than the similarity between random pairs of parcels; however, it decreases during the gestation (C). This analysis was repeated for all parcels of all fetuses and the test-statistics (sorted by age) is shown in (D). *P*-values of the estimated slopes for decreasing similarity during gestation is demonstrated in (E).

The similarity of mirrored functional connectivity profiles between homologous parcels was significantly higher than the similarity between random pairs of parcels (P<0.05 after FDR correction) in the entire population. It was quantified as Pearson’s correlation coefficient between functional connectivity profiles (vectors of edge values after thresholding negative and weak connections) of two parcels (Fig. 4A). For a given parcel of a single fetus, we first tested if its similarity to its contra-lateral homolog is significantly higher than its similarity to all other parcels (Fig. 4B). The test was repeated for all parcels of all fetuses and the assumption was held for all except in six instances (temporal-Inferior (1 fetus, GW27^w^+3^d^), Rectus (1 fetus, GW29^w^+3^d^), Heschl (2 fetuses, GW31^w^+6^d^ and GW32^w^+4^d^), Temporal-Pole-Sup (1 fetus, GW^w^+2^d^)). The resulting test statistics sorted by age are shown in the Fig. 4D, where the regular increase of the statistic values indicates similarity decreases with advancing gestational age.

### Lateralization of the functional connectome

**Fig. 5:**
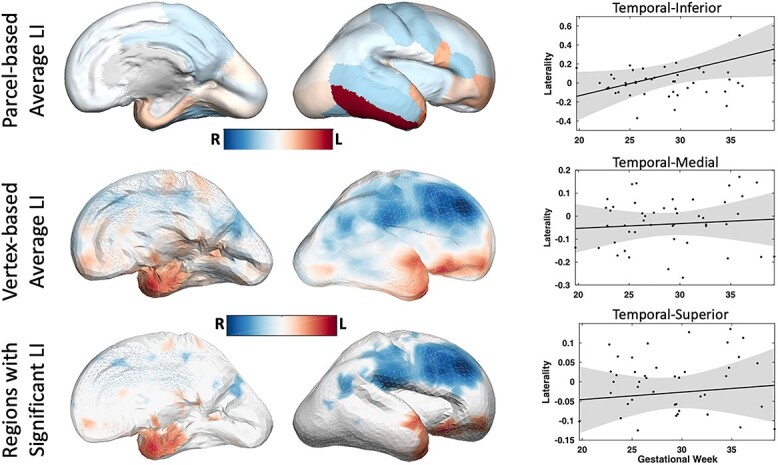
Multi-subject maps of brain functional asymmetry. Image-analysis techniques make it possible to distinguish systematic functional asymmetries in a population, or a specific group of subjects, from random fluctuations in functional connectivity. Average LI values across 48 normal fetuses are shown in color which reveals prominent asymmetries in Temporal-Inferior, Temporal-Medial, and Temporal-Superior areas. By comparing the average magnitude of these asymmetries with their standard error—derived from the standard deviation—regions of significant asymmetry are identified.

We first tested if there was significant functional lateralization of connectivity in any cortical regions averaged over the entire studied prenatal period. Among all cortical regions, we found significant functional lateralization in Superior-Temporal (*P*=0.043, tstat: -2.071, CI=[-0.0567,-0.00082], sd=0.096), Medial-Temporal (*P*=0.047, tstat: -2.030, CI=[-0.0691,-0.00032], sd=0.118), and Inferior-Temporal (*P*=0.048, sd=0.311, tstat: 2.031, CI=[0.0008,0.1818]) regions. Figure 5 shows the average lateralization index, on both parcel- and vertex level for the regions with significant laterality as color-coded maps. Next, we investigated how functional laterality develops in those temporal regions with a linear regression model. Only for the Inferior-Temporal region, there was a significant increase in functional connectivity laterality with gestation age (slope: $0.025\pm 0.0087$, *P*=0.005) with the adjusted R$^2$ of 0.156. No significant pattern between age and functional laterality was found in Superior-Temporal and Medial-Temporal regions.

## Discussion

This study presents the first in-vivo analysis of the development of the functional thalamocortical connectome and the associated cortico–cortical connectivity in living human fetuses before birth. It contributes new pieces of evidence for the timing and heterogeneity of functional development accompanying the parallel structural development observed in ex-vivo studies. In-utero rs-fMRI in normal fetuses from 19 to 39 weeks of gestation revealed a symmetric sigmoid development of thalamocortical connectivity with peak increase of connection strength between 29^th^ and 31^st^ weeks of gestation (27–29 PCW). This is a period associated with the massive growth of axons into the cortical plate and with the proliferation of dendrites (Molliver et al. 1973; Kostovic and Rakic 1990; Kanold and Luhmann 2010). It was accompanied by a strengthening of cortico–cortical functional connectivity and significant functional laterality in temporal-superior, -medial, and -inferior regions. Homologous contra-lateral cortical areas exhibited significant similarity in their mirrored connectivity development. At the same time, this similarity decreased during gestation, suggesting an increasingly diverse connectivity landscape of the two hemispheres after initial symmetry.

### Developmental trajectories of thalamocortical functional connectivity revealed by fMRI

The establishment of functional thalamocortical connections for different parcels in the occipital lobe revealed a nonlinear sigmoid pattern of strengthening as a function of gestational age independent of the distance between parcels. The inflection points corresponding to the maximum increase of connectivity strength occur from 29^th^ and 31^st^ weeks of gestation. This coincides with a period of densely innervation and penetration of thalamocortical afferents into the cortical plate as revealed by acetylcholinesterase (AChE) histochemistry and immunohistochemical methods on postmortem fetal brains (Kostovic and Rakic, 1984b, 1990; Kostović and Judaš 2006; Krsnik et al. 2017; Wang et al. 2017). These studies found that during this period lateral geniculate nucleus (LGN) thalamic axons are on the verge of establishing synapses with neurons of layer IV of the primary visual cortex and create a structural substrate underlying sensory-expectant activation and functional connectivity. In particular, early in development, ocular dominance columns (ODCs) in layer IV of visual cortex are absent and the projection of LGN thalamic axons representing each eye are initially diffuse, imprecise, and overlapping (Rakic 1976; Mooney et al. 1996; Penn and Shatz 1999; Sherman and Guillery 2013). Since retinal gangelion cells are spontaneously active in the dark (even before the development of retinal photo-receptors in utero (Mastronarde 1989)), they can endogenously generate highly correlated bursts of action potentials that drive the strengthening, refinement, and remodeling of the early synaptic connections into the precise adult-like circuits. The formation of the dense and eye-specific adult-like pattern of synaptic circuitry gives rise to the observed coordinated pattern of neuronal activity and functional connectivity. The observed emergence of functional synchronization before birth complements observations in primates, where orientation columns and ODC are already forming in utero before visual experience (Katz and Shatz 1996; Penn and Shatz 1999; Horton and Hocking 1996). Animal experiments have indicated that blocking this correlated neural activity or biasing the competition in favor of one eye results in a severe disruption of the ultimate pattern of connections in visual cortex (Penn et al. 1998; Stryker and Harris 1986; Katz and Shatz 1996; Balice-Gordon and Lichtman 1994).

A second potential contributor to the early observed synchronous oscillations in the form of functional connectivity is the subplate, a transient population of early-born neurons that receive thalamic input and make synaptic connections with the overlying cortex (Kostović and Judaš 2010; Kanold and Luhmann 2010). In animal experiments, it has been shown that this transient fetal circuitry of the subplate zone is functional, and involved in the formation of ODCs and orientation selective response in visual cortex (Kanold et al. 2003). In humans the subplate develops to approximately four times the thickness of the cortical plate around 29 weeks of gestation and occupies up to 45% of entire telencephalic volume (Vasung et al. 2016). It was suggested that the functional circuitry of the voluminous subplate is a significant player in shaping cortical activity in the preterm infants (Kostović 2020). Thus, our findings of the establishment of synchronized functional activity as early as 29–31 GWs are in line with the period of maximum volume of subplate and increased synaptic interactions with neurons of layer IV.

In addition, the final asymptotic phase of the thalamocortical trajectories might indicate the stabilization of the functional connectivity by the end of gestation and reaching the full capacity that a developing brain can serve during the prenatal period. This phase is also related to the period of the dissolution of subplate and consolidation of the existing connections (Kostović and Judaš 2010; Kanold and Luhmann 2010). Further refinement of these connections might occur postnatally when external visual stimuli are perceived.

In contrast to the visual cortex, parcels in parietal- and frontal areas revealed accelerating, complex nonlinear trajectories as a function of both age and distance from the thalamus to the parcels. Instead of a plateau as in the visual cortex, they lack convergence to a stable value and exhibit variable growth rate at the last weeks of gestation. This suggests that in these regions functional development is either ongoing or just starting during late gestation. In these parcels the inflection points of maximum connectivity increase were most variable and were distributed across a broad range of gestational weeks. The histochemical and morphological maturation of the mediodorsal nucleus (MD) of the thalamus and and its connections to the frontal lobe also revealed a more prolonged timetable of thalamocortical outgrowth as ChE staining reaches peak intensity in MD between 22 and 30 weeks of gestation^1^ and is correlated in time with the appearance of laminar-specific ChE staining in the frontal cortex between 26 and 32 weeks of gestation (Kostovic and Goldman-Rakic 1983; Kostovic and Rakic 1990). However, ingrowth of fibers to the somatosensory cortex seems to precede ingrowth to the frontal and occipital areas for almost 2 weeks (Kostović and Judaš 2006; Krsnik et al. 2017). Overall, the interaction between thalamocortical axons and subplate neurons during prolonged midfetal growth (lasting 4 months) is crucial for establishing early transient fetal functional circuitry (Kostovic and Rakic 1990; Kostović and Judaš 2007; Kostovic and Judas 2009). Based on the existing evidence, we propose that the critical period for the functional development of the thalamocortical connectivity exists between 24 and 31 gestational weeks, in which normal patterns of neural activity and synaptogenesis within the transient subplate zone are necessary for the formation of the adult pattern (Molliver et al. 1973; Kostovic and Goldman-Rakic 1983; Kostovic and Rakic 1990; Shatz 1992). Insults during this developmentally critical period may promote the recruitment of alternative axonal pathways to sustain function (Uylings 2006), affecting the specification and integration of functional circuits at both cortical and sub-cortical levels (Crair and Malenka 1995).

### Development of the functional cortio-cortical connectome revealed by fMRI

Our results confirm the presence of the macro-scale functional cortico–cortical circuitry is best described with a sigmoid model (mean adjusted $r^2$ ranging from 0.55 to 0.74, Fig. 3 showing cortico–cortical connectivity) and emerges in the same gestational period as the thalamocortical functional connectome. Again, the significant rise in the maturation of the lobar functional connectivity at the transition from the late second to the third trimester with the peak around the 29th GW (Fig. 3) was in line with histological descriptions of *ex-vivo* fetal tissue and the underlying synaptogenesis in humans (Kostović and Judaš 2006, 2010; Krsnik et al. 2017; Vasung et al. 2016). In particular, the appearance of synapses, dendritic arborization, and axonal tracings (Huang and Vasung 2014; Kostović and Judaš 2006, 2007; 2010; Vasung et al. 2017), as well as ex-vivo human fetal DTI studies on the axonal connectivity (Huang et al. 2006, 2009; Takahashi et al. 2012; Vasung et al. 2010, 2017; Wang et al. 2017) showed during the early fetal period (11–17 GW), several limbic bundles can be already recognized (e.g. fornix, stria terminalis, and cingulum). The appearance of limbic bundles is followed by the accumulation of afferent thalamocortical fibers in the intermediate and subplate zone (17–25 GW), and the appearance (26–34 GW) and completion of associational fiber bundles (35–40 GW). In parallel, fetal functional maturation in the form of the development of sensorimotor functions (e.g. maturation of primary cortices and projection fibers), emotional development (e.g. maturation of limbic system), and establishment of higher order cognitive skills (e.g. maturation of prefrontal cortex and association fiber bundles) is progressed (Behzadi et al. 2007; Vasung et al. 2019, You et al. 2016). Transient circuits in the fetal brain, which show endogenous and spontaneous activity and are centered around the subplate zone, are gradually replaced by adult-like, sensory-driven ones (Kostović and Judaš 2006, 2007).

Recent fMRI findings in prematurely born infants and fetuses showed the presence of fundamental properties of the neural connectome, including a functional small-world, modular, and rich-club architecture as early as the third trimester (Turk et al. 2019; Van Den Heuvel et al. 2015). These fMRI studies relied on the spatial normalization of the fMRI volumes to a standard average 32-week brain template for group comparisons resulting in averaging across a wide range of gestational ages. Taking the dramatic developmental processes of the brain even within 1 week of gestation into consideration, we used an age-specific fetal template (Gholipour et al. 2017) providing a consistent set of 78 cortical regions (39 regions per hemisphere) which could capture the anatomical variability of the fetal population and allow cross-sectional assessment of the FC across gestation. Unlike the study of (Jakab et al. 2014), our results did not reveal a significant maturational sequence of inflection times of functional development of the cortical lobes. In contrast to that study, we analyzed connections to all other parcels, instead of functional connectivity within lobe networks. However, the difference of a narrow window of sigmoid inflection followed by a plateau in the visual cortex compared with more variability and continued increase in frontal-, parietal areas resembles differences observed in (Jakab et al. 2014).

### Functional similarity and laterality

This is the first study evaluating the development of functional laterality and similarity in the developing brain of fetuses in utero. The similarity of the mirrored connectivity profiles was significantly higher for contra-lateral homologous regions compared with random pairs of the regions. At the same time, this similarity decreases with advancing gestational age (Fig. 4C) suggesting an increasingly complex lateralized integration of circuitry across the cortex during this phase. Functional connectivity in the inferior-, medial-, and superior-temporal regions corresponding to Wernicke’s area was significantly left-lateralized with laterality increasing during the late second and third trimester of gestation (Fig. 5). Although we did not find significant functional laterality in any other brain regions including Broca’s area, our results are in line with studies suggesting the brain is primed for language already before birth and the receptive language precedes expressive language (Lebenberg et al. 2019; Can et al. 2013). A recent longitudinal study of preterm born infants and the changes of inter-hemispheric connectivity from 30 gestational week to the first months of life demonstrated that the rate of increase of inter-hemispheric connectivity of the Wernicke’s homologs is significantly greater than that of the Broca’s homologs (Scheinost et al. 2022).

The relationship between the observed functional laterality and structural development of the brain is particularly interesting. Anatomical development clearly shapes and imposes constraints on brain function, and in turn can be modified by functional activity. Structural brain asymmetries have been identified and reported by anatomical post mortem and prenatal MR studies as earlier emergence and deeper sulcal pit of the right superior temporal sulcus (Kasprian et al. 2011; Habas et al. 2012). Moreover, Schwartz et al. found a significant reduction of the cerebral wall thickness and a significantly smaller structural asymmetry index in temporal regions of the fetuses with complete agenesis of corpus callosum compared with healthy controls (Schwartz et al. 2021). In other words, normal development of the brain imposes significant structural asymmetry in the temporal region and our observation of significant functional laterality and decreased similarity in this area show another evidence for interrelationship between structure and function. Although, clarifying the the exact nature of this relationship is beyond the scope of this work.

### Limitations

This study has several limitations. Angiogenesis can influence the vascular contribution to the BOLD signal but detailed information about this developmental coupling is as of yet unavailable. The changing tissue composition throughout gestation potentially introduces varying levels of partial volume effects in structural imaging. Thus, fetal growth, itself, interacts with MRI imaging of anatomy. One of the biggest challenges in the analysis of fetal MRI is motion originating both from spontaneous fetal motion and from maternal movement. Although we corrected motion and compensated for associated signal variation, there remains the possibility that motion artifacts are still present in the analyzed data (Sobotka et al. 2022). Finally, the voxel size in fetal fMRI and rs-fMRI studies have 2–4 mm thickness (Jakab et al. 2014; Thomason et al. 2014) and therefore the delineation of subplate and cortex in fMRI is limited. Thus, separate analysis of spontaneous activity of fetal circuitry and activity that is sensory driven, using in-utero MRI, still remains elusive.

## Conclusion

Brain development during fetal life provides a foundation for short- and long-term neurobehavioral outcomes, making the prenatal phase probably the most important period of our structural and functional growth and maturity. There is growing evidence that neuropsychiatric disorders like schizophrenia, autism, and bipolar disorder have their origin in abnormal development of the brain during gestation. Thus, establishing a baseline for normal development of the thalamocortical circuits in early life is clinically relevant to understanding which characteristics of thalamocortical development may be selectively vulnerable to injury and leads to neurodevelopmental disorders. A better understanding of brain functioning at this period may pave the way for early detection of risks and clinically significant anomalies. In addition, considering the extreme plasticity of the developing brain, it might present a window of opportunity for early intervention and redirecting aberrant developmental trajectories back onto a normal pattern.

## Supplementary Material

cercor_supplementary_bhac446

SourceFiles_bhac446

## Footnotes

1 The gestational ages in these papers are expressed as the gestational weeks from ovulation but it is reported here from the first day of the last menstrual period.
