# Supplementary material for: Fetal development of functional thalamocortical and cortico–cortical connectivity
Source: Cereb Cortex. 2022 Dec 15;33(9):5613–24. doi: 10.1093/cercor/bhac446 (PMC10152101; doi:10.1093/cercor/bhac446)
Supplement: cercor_supplementary_bhac446 [file cercor_supplementary_bhac446.pdf]

Supplementary Information

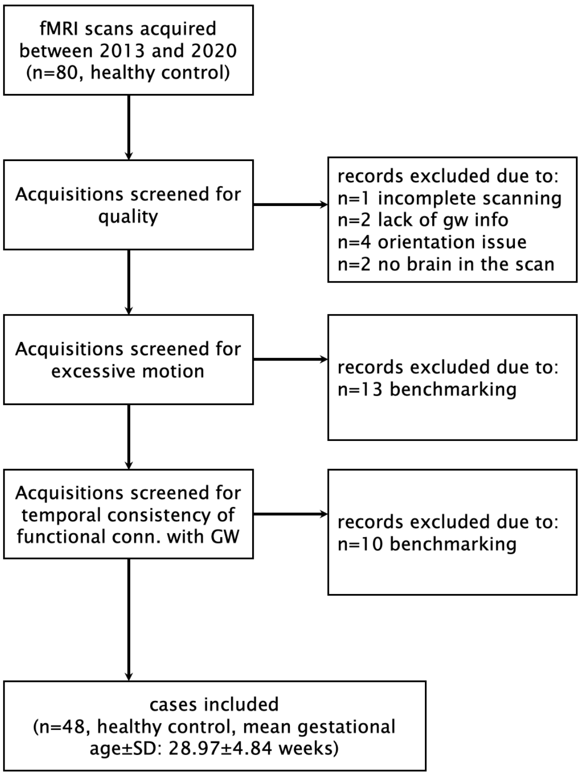

Figure S1: Schematic depiction of the case-selection procedure employed in this study.

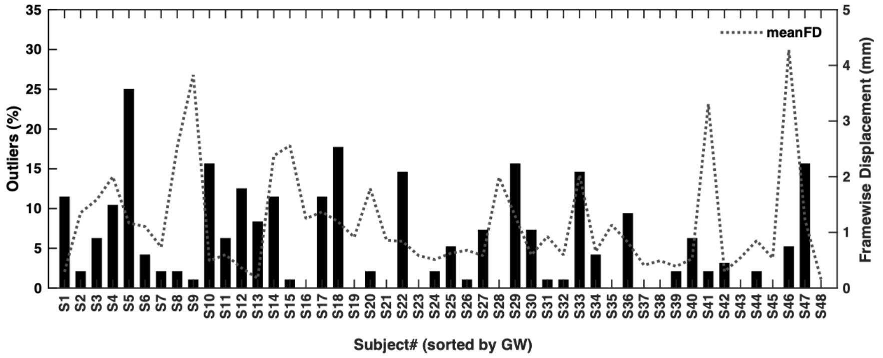

Figure S2: The percentage of the outlier volumes (left y-axis) and the average frame-wise displacement (right y-axis) of the final cohort of subjects employed in this study. The average percentage of outlier volumes in the rs-fMRI time series for the remaining subjects was 6.14% (range:0-25%). It was marginally correlated with gestational age ($R = -0.26, P = 0.048$) and significantly decreased after our method of motion correction compared to the original data ($P = 0.032$). Fetal head movements quantified by either the mean or the maximum of the frame-wise displacement didn't correlate with fetal age ($P = 0.56$ and 0.26 respectively).

Structural Data Processing

Preprocessing of T2 images consisted of denoising (?), in-plane super-resolution (?), and automatic brain masking (?). For each subject, we had between three and seven T2 images in 3 orthogonal directions. The orientation of each scan was determined based on the location of the bodily organs of the fetus. The pre-processed scans were combined into a single 0.5 mm isotropic volume using a state-of-the art, combined, slice-wise motion correction and volumetric super-resolution algorithm (?). The resulting volumes were then rigidly aligned to a common reference space (?). Segmentation of the developing cortical plate, brain pallium, as well as the lateral ventricles in the isotropic volumes, was performed by nonrigid mapping of the Gholipour atlas (?) to the individual cases. To account for variability in development, as well as limits in the accuracy of estimating the exact date of conception, atlases that spanned the prior and consecutive weeks of the estimated gestational age of each case were also used and merged using a label fusion technique (?). The quality of the resulting segmentations was checked (but not manually corrected) at this stage and after registering to the individual fMRI space. Overall, 78 cortical regions of interest (ROIs) listed below and six subcortical structures including the thalamus were consistently found in all subjects.

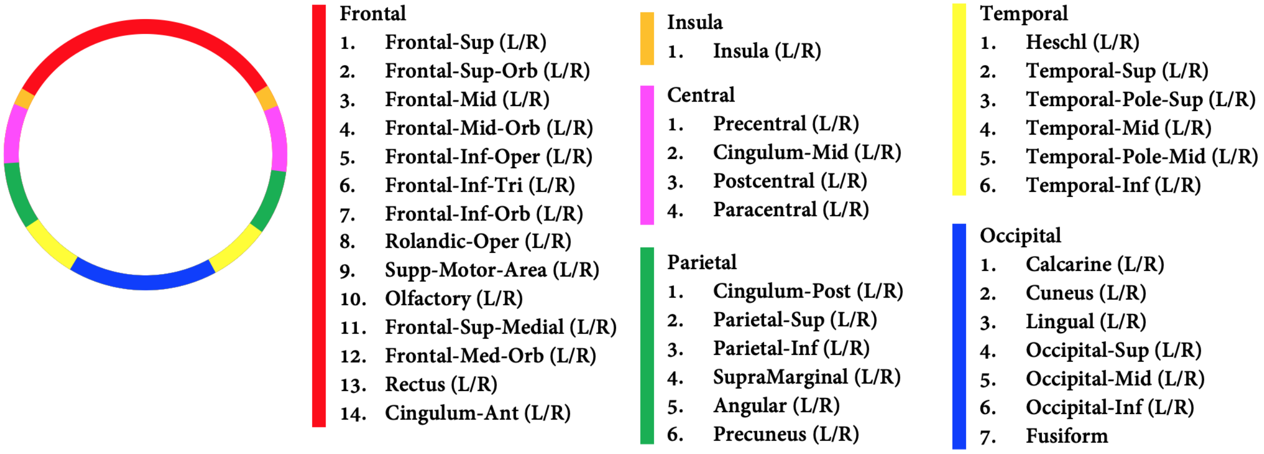

Figure S3: Derived Cortical Regions of Interest.

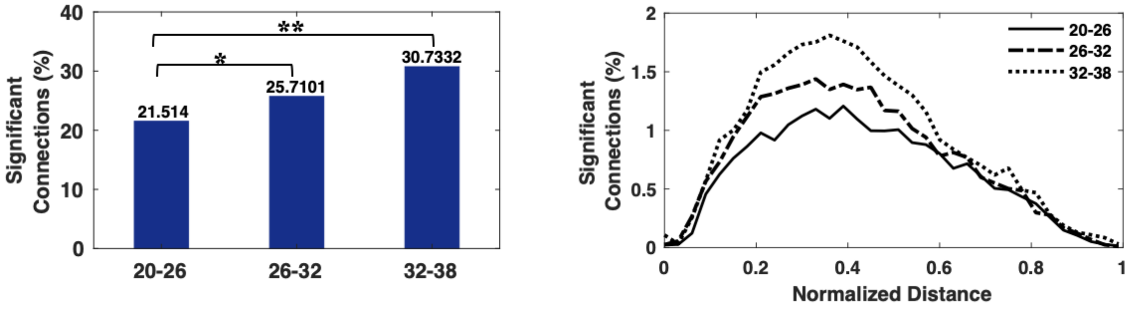

Figure S4: Overall age-dependent growth pattern of functional connections. left: the connection density plot of all three age groups (based on statistical testing of connections (P<0.05, FDR corrected)); right: the histogram plots of the normalized anatomical distance associated with the significant connections for each age group.

Analysis of the robustness:

We have conducted additional experiments to assess the stability of results when in addition to the employed benchmarking strategies, the percentage of outlier volumes is also thresholded, and thus less subjects are included in the analysis. We set a threshold of the maximum of 10% outlier volumes which led to the exclusion of 12 additional subjects from the final analyzed cohort in the study. The average gestational age of this remaining cohort with 36 subjects was 29.65±4.7 weeks and the average number of outlier volumes was 2.52±2.66. Neither the maximum nor the mean frame-wise displacement was significantly correlated with gestational age (p-values equal to 0.29 and 0.39 respectively).

We examined the robustness of our finding that the similarity between the connectivity profile of the homologous regions decreases with advancing gestational age based in the new reduced cohort. Note that in the paper, we first showed for each subject the similarity between contra-lateral homologous regions is significantly higher than the similarity between random pairs of regions, and this finding is subject-specific and does not change even with excluding more subjects. On the reduced size cohort, the similarity between hemispheres connectivity profiles decreases, but for some regions, this decrease is no longer statistically significant. For instance, the p-value of the estimated slope of the similarity changes in the Supramarginal region has shifted from 0.003 for the original cohort to 0.0893 for the new reduced cohort of subjects. Other areas such as Precuneus and Calcarine continue to show a significant decrease in similarity. Figure.S5.A shows a visualization corresponding to figure.**??**.E in the manuscript on the areas with significantly decreasing similarity for the reduced size cohort (10% cut-off) .

We also evaluated the robustness of our finding of the developmentally increasing functional laterality using the new reduced size cohort of subjects. All the three areas in the temporal region with significant functional LI were replicated (see figureS5.B for temporal inferior region with p-value of 0.04). The robustness of the developmental models of thalamocortical and corticocortical connectivity was investigated in the main paper using the bootstrapping technique. Overall this analysis suggests that the findings are stable, but that the increased cohort size with a more tolerant outlier cut-off affords more data to identify changes in laterality and similarity correlated with gestational age.

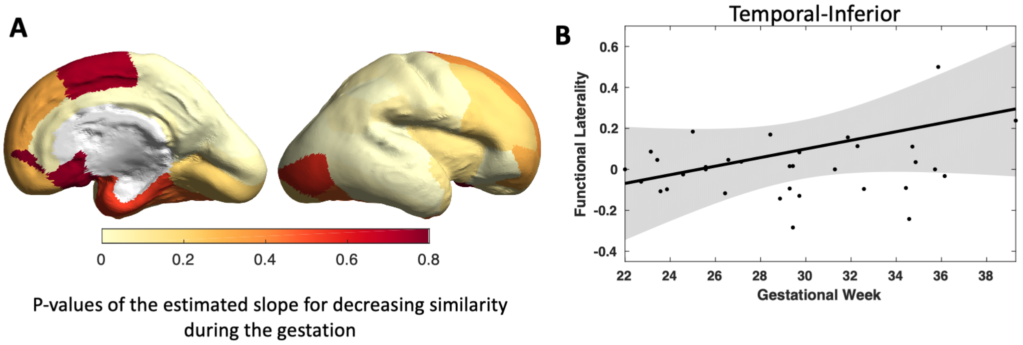

Figure S5: Analysis of the robustness. left: brain areas with significant similarity decrease and right: the increase of functional laterality in the reduced size cohort (10% cut-off of outlier volumes).

Inflection time:

The figure below shows an example of the development of thalamocortical functional connectivity in the left-middle-occipital region. Each black dot in the figure represents a single subject. We observed the strength of the functional connectivity in this region starts at around zero for the younger fetuses and becomes stronger for the older ones. Since the development of functional connectivity is an asymptotically bounded process in general, we thought the sigmoid function could be a good candidate for modeling the observed developmental process allowing us to summarize it in terms of initial (β1) and final (β2) quantity, as well as the inflection time (β3) and the rate (β4) of its growth. The first derivative of the sigmoid function is bell-shaped and shows how fast the observed process changes with advancing time (dashed line). The local maximum of the bell represents the inflection point at which the growth of the functional thalamocortical connectivity is maximal. We estimated the parameters of the model using robust Least Absolute Residual and assessed their significance level by testing the null hypothesis that each individual parameter is equal to zero. In addition, a bootstrap approach was utilized to account for the sampling variability in which 75% of the subjects were selected randomly, and the fitting of the sigmoid function and the estimation of the parameters was repeated 1000 times. The distribution of the achieved inflection times in this way was also depicted in the following figure (and in the paper). We hypothesized that if the neurons of a brain region are connected to each other, a synchronized neural activity is observed in that region (cells that wire together fire together).

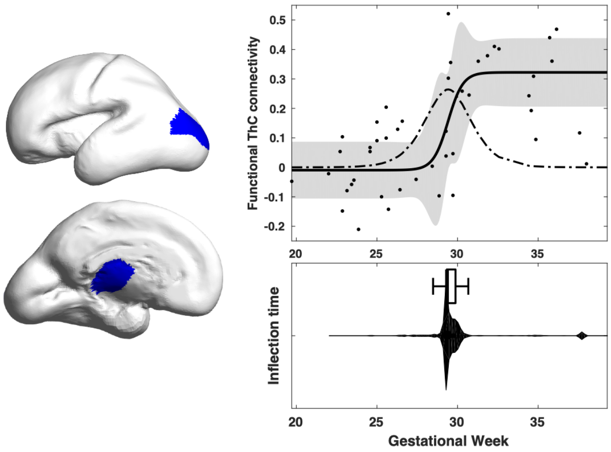

Figure S6: A detailed description of the inflection time and the curve-fitting was conducted in this study.
