# Supplementary figures and images for: Fetal development of functional thalamocortical and cortico–cortical connectivity
Source: Cereb Cortex. 2022 Dec 15;33(9):5613–24. doi: 10.1093/cercor/bhac446 (PMC10152101; doi:10.1093/cercor/bhac446)

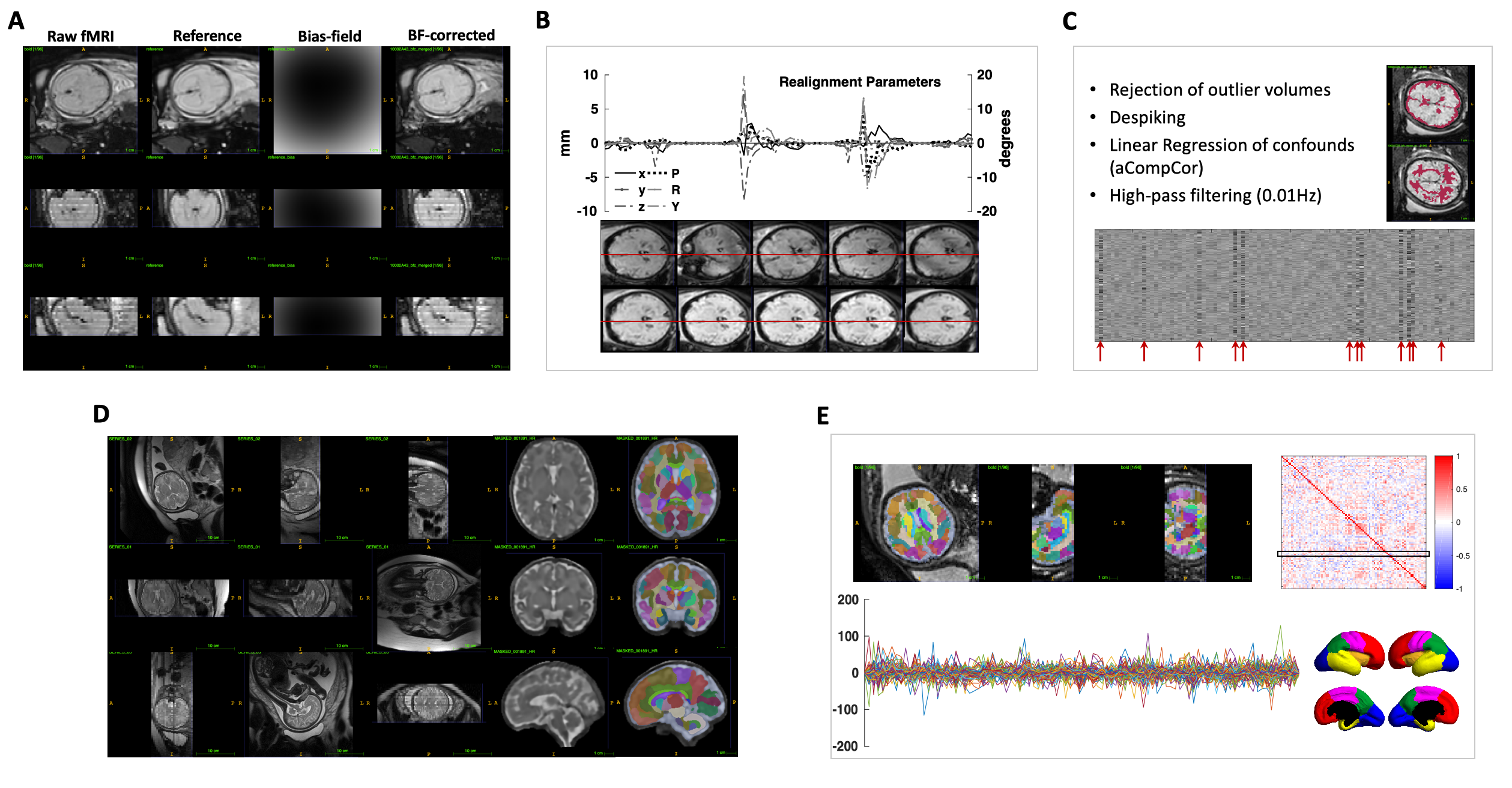

Supplement: SourceFiles_bhac446 [file sourcefiles_bhac446.zip › Figures/workflow.png]

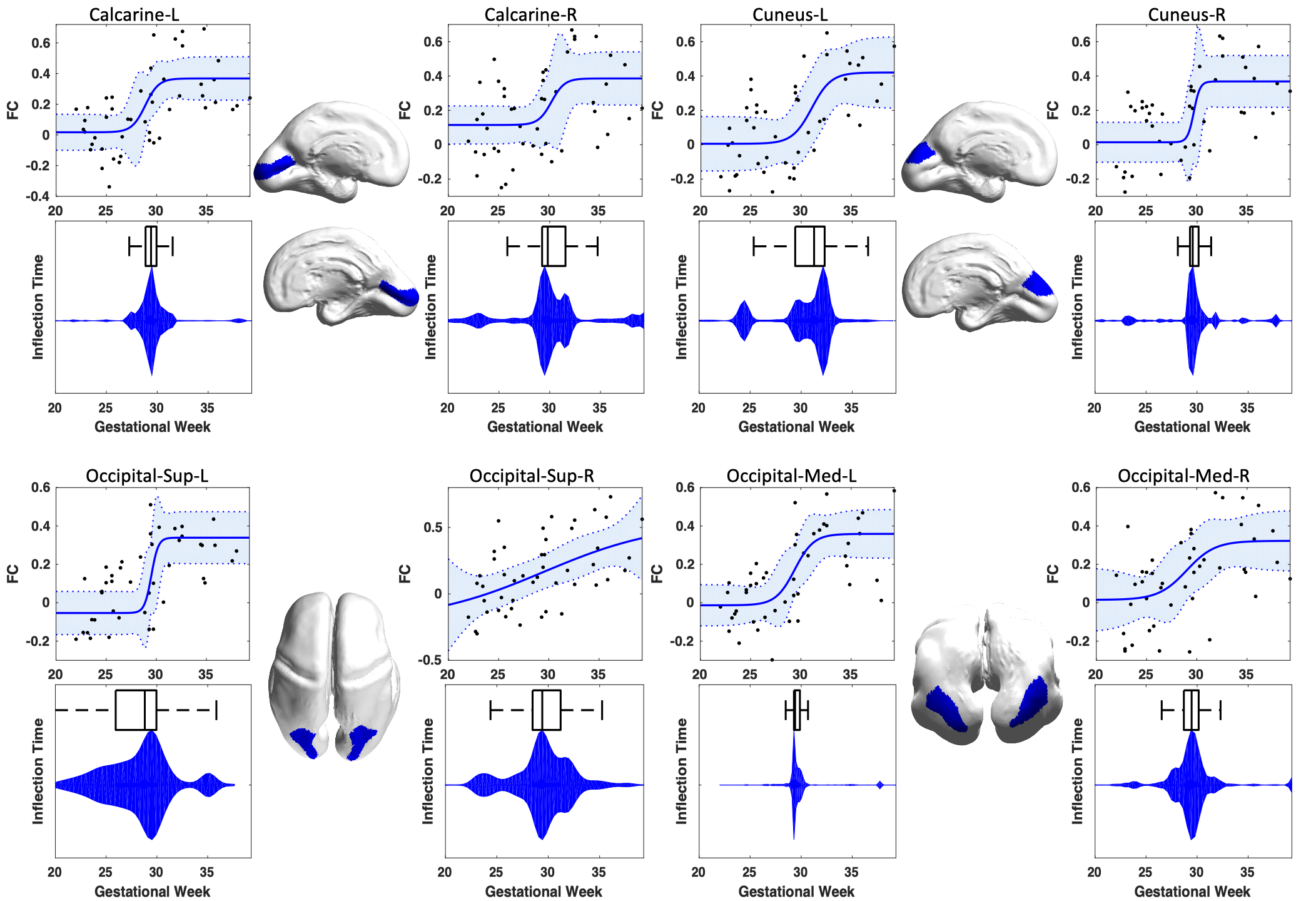

Supplement: SourceFiles_bhac446 [file sourcefiles_bhac446.zip › Figures/ThC-Visual.png]

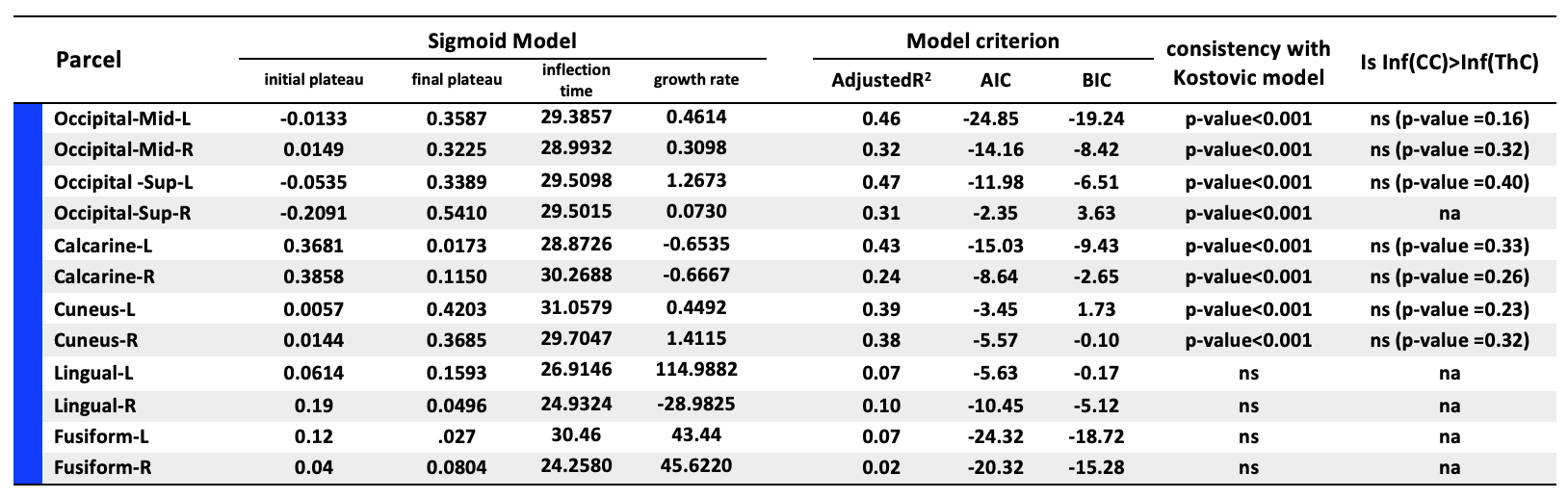

Supplement: SourceFiles_bhac446 [file sourcefiles_bhac446.zip › Figures/TableS1.png]

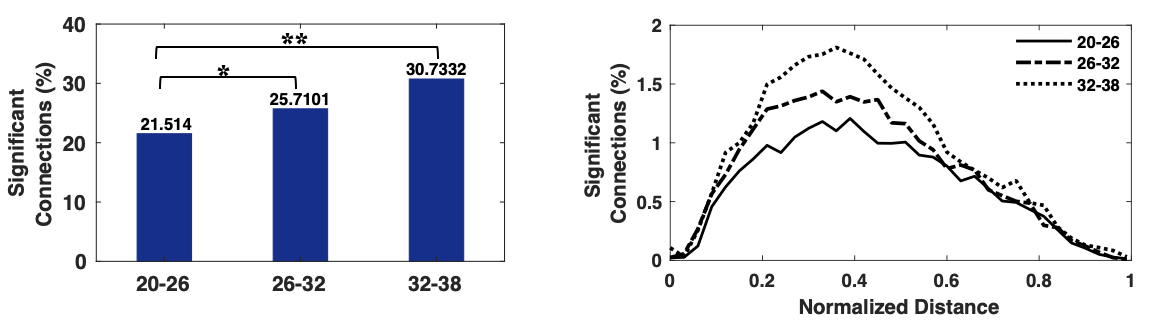

Supplement: SourceFiles_bhac446 [file sourcefiles_bhac446.zip › Figures/Overall.png]

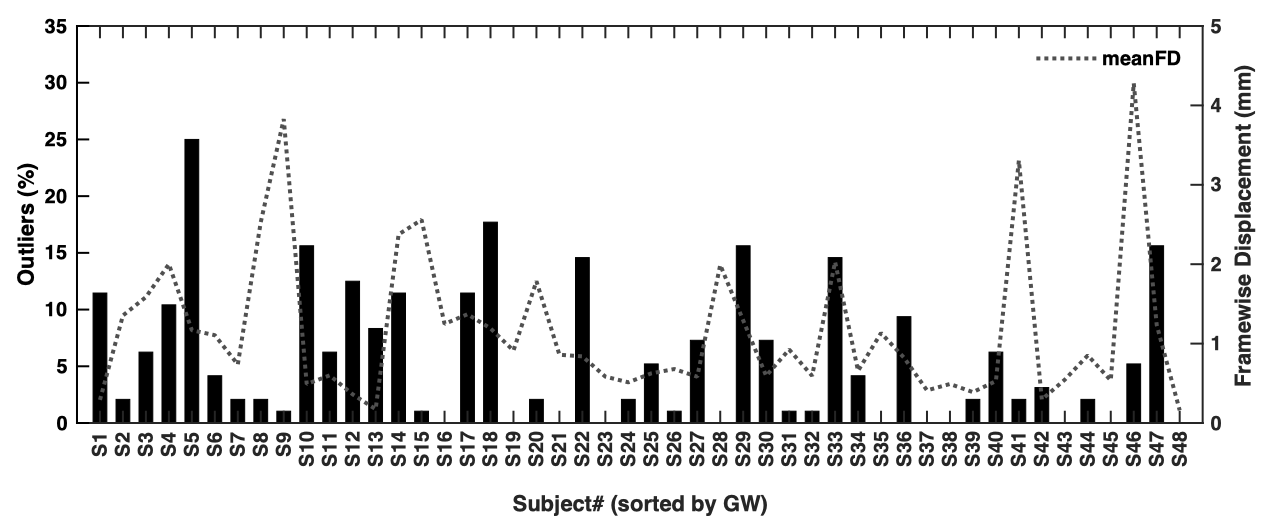

Supplement: SourceFiles_bhac446 [file sourcefiles_bhac446.zip › Figures/Outliers.png]

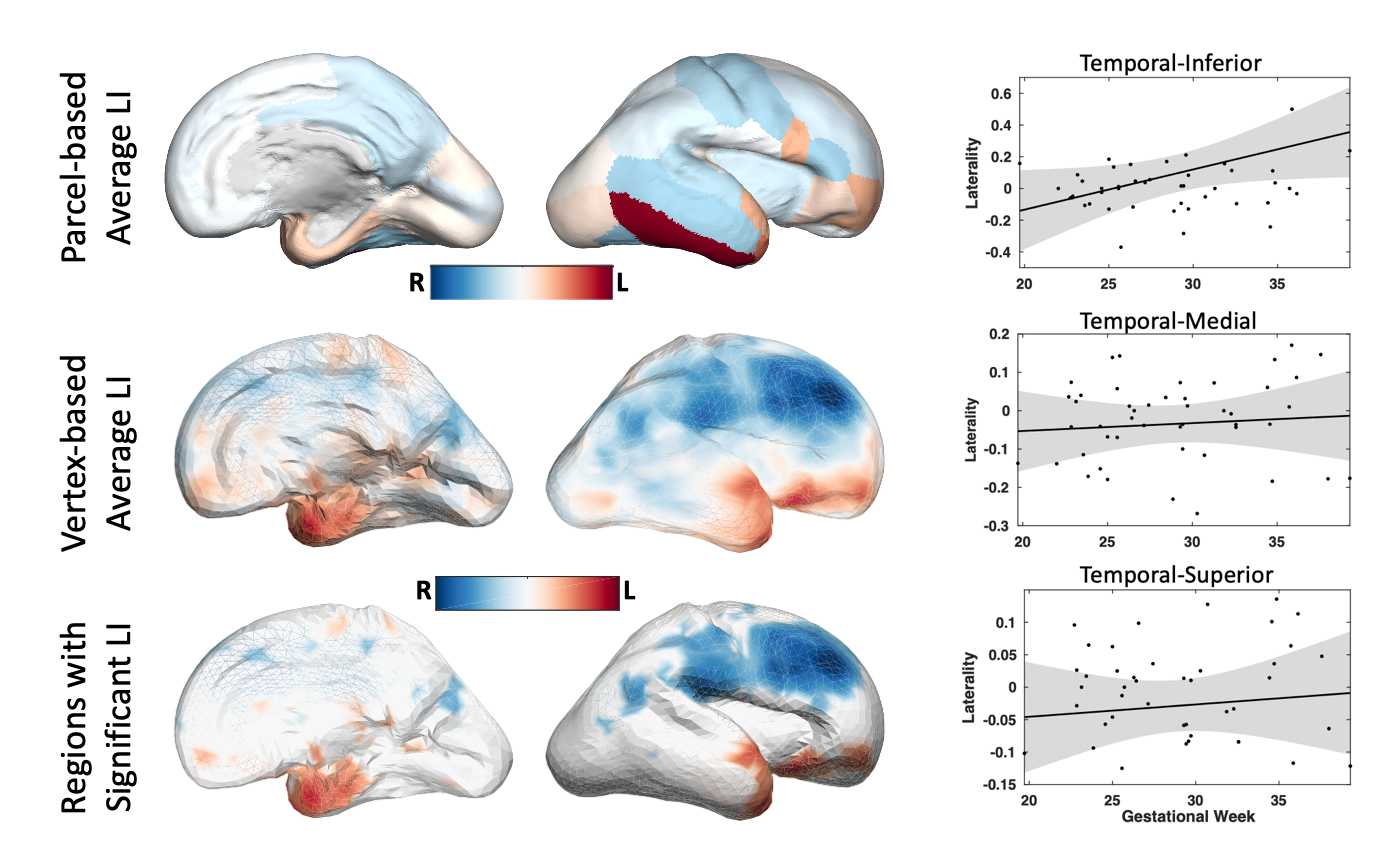

Supplement: SourceFiles_bhac446 [file sourcefiles_bhac446.zip › Figures/LI.png]

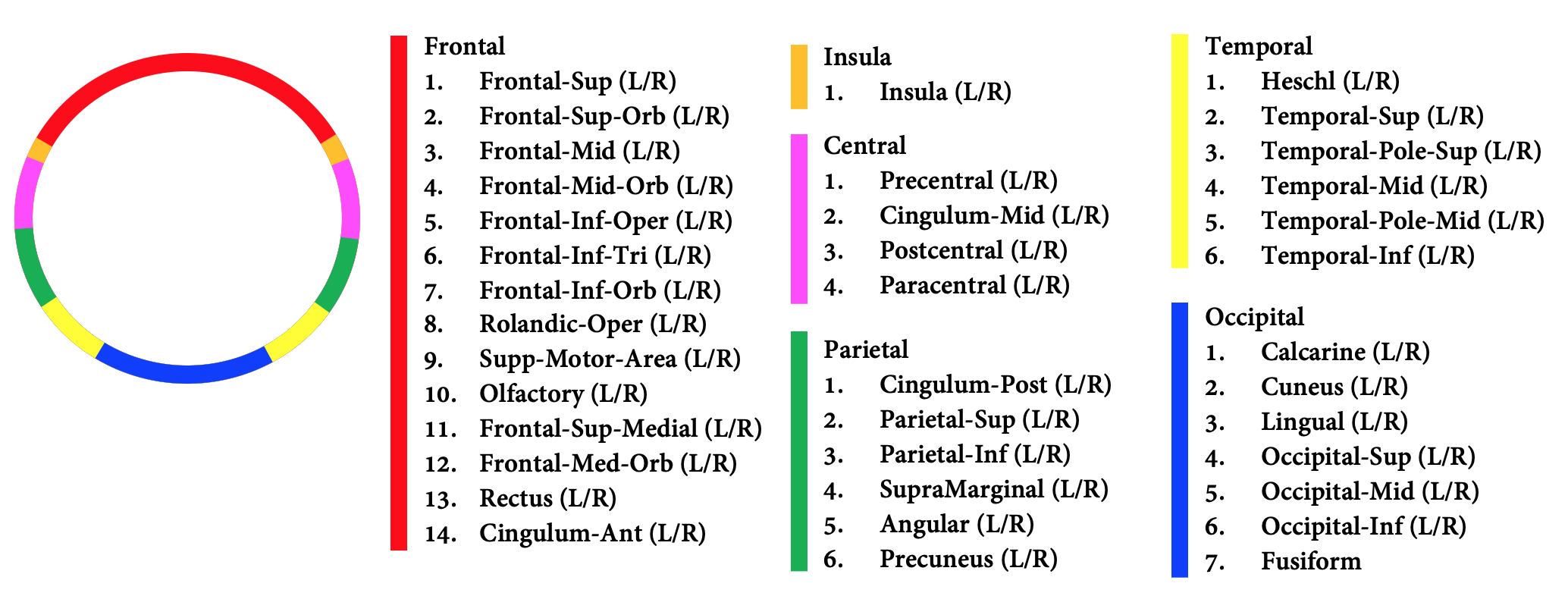

Supplement: SourceFiles_bhac446 [file sourcefiles_bhac446.zip › Figures/cortical-labels.png]

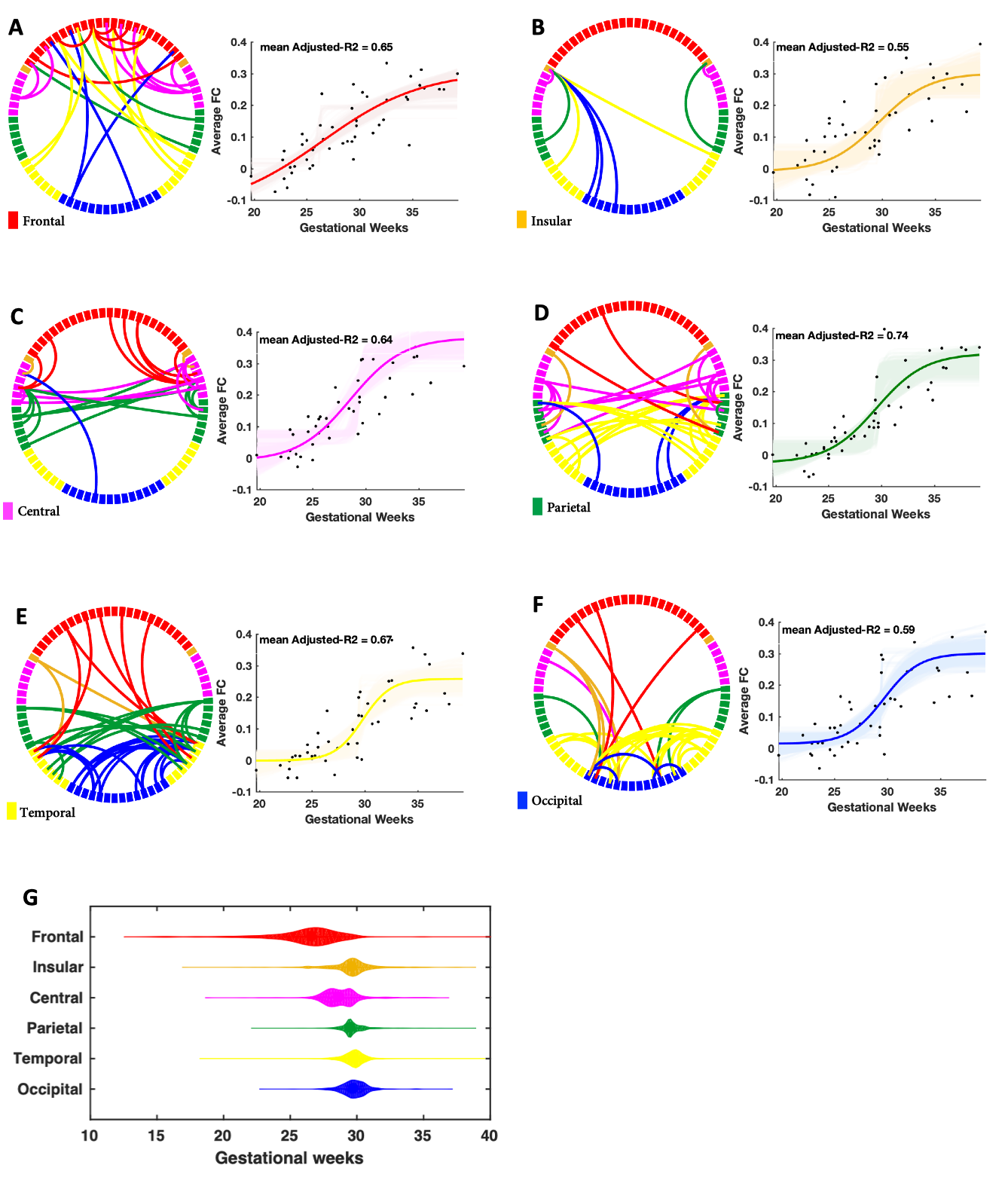

Supplement: SourceFiles_bhac446 [file sourcefiles_bhac446.zip › Figures/CCTrajInf.png]

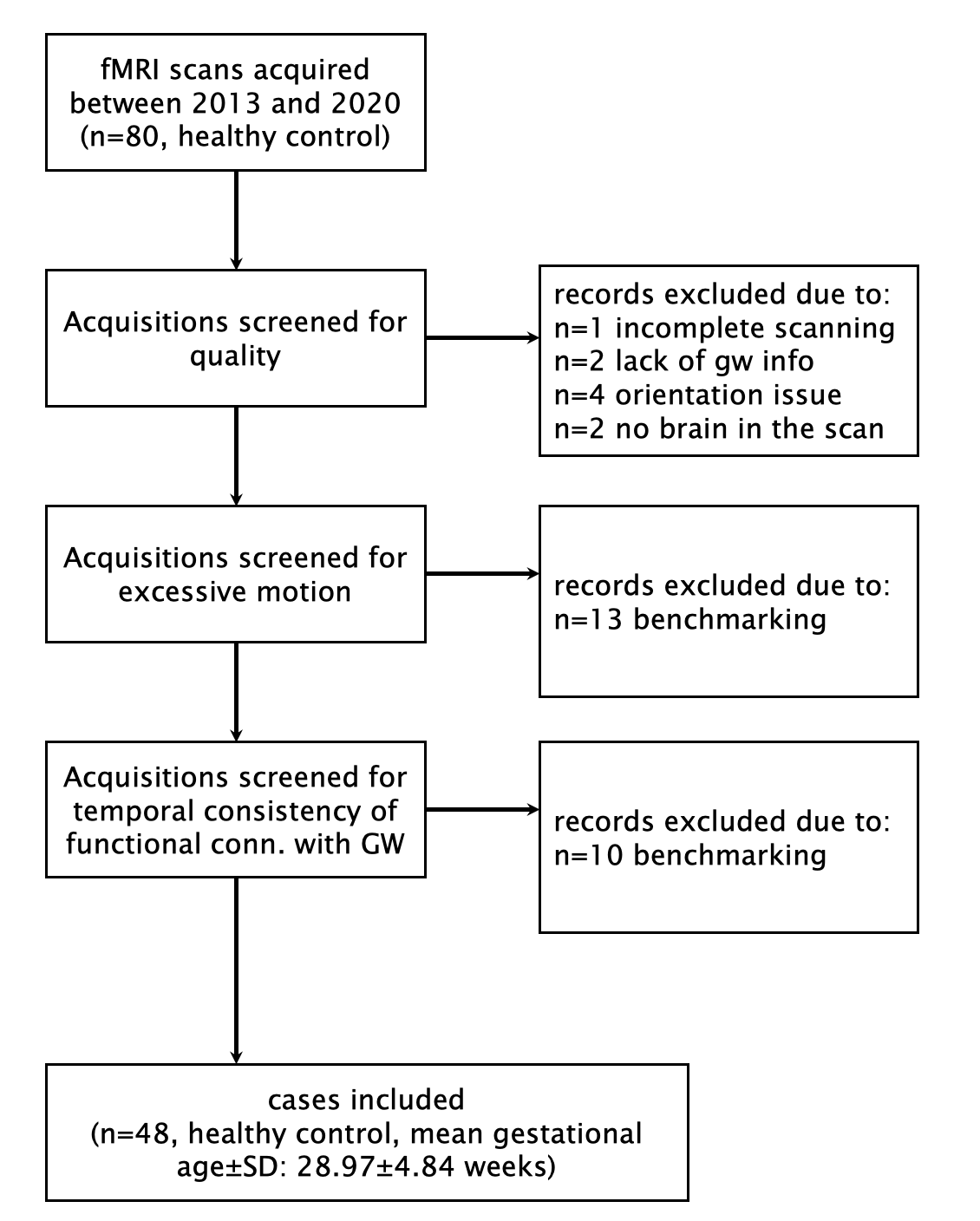

Supplement: SourceFiles_bhac446 [file sourcefiles_bhac446.zip › Figures/Case-selectionProcedure.png]

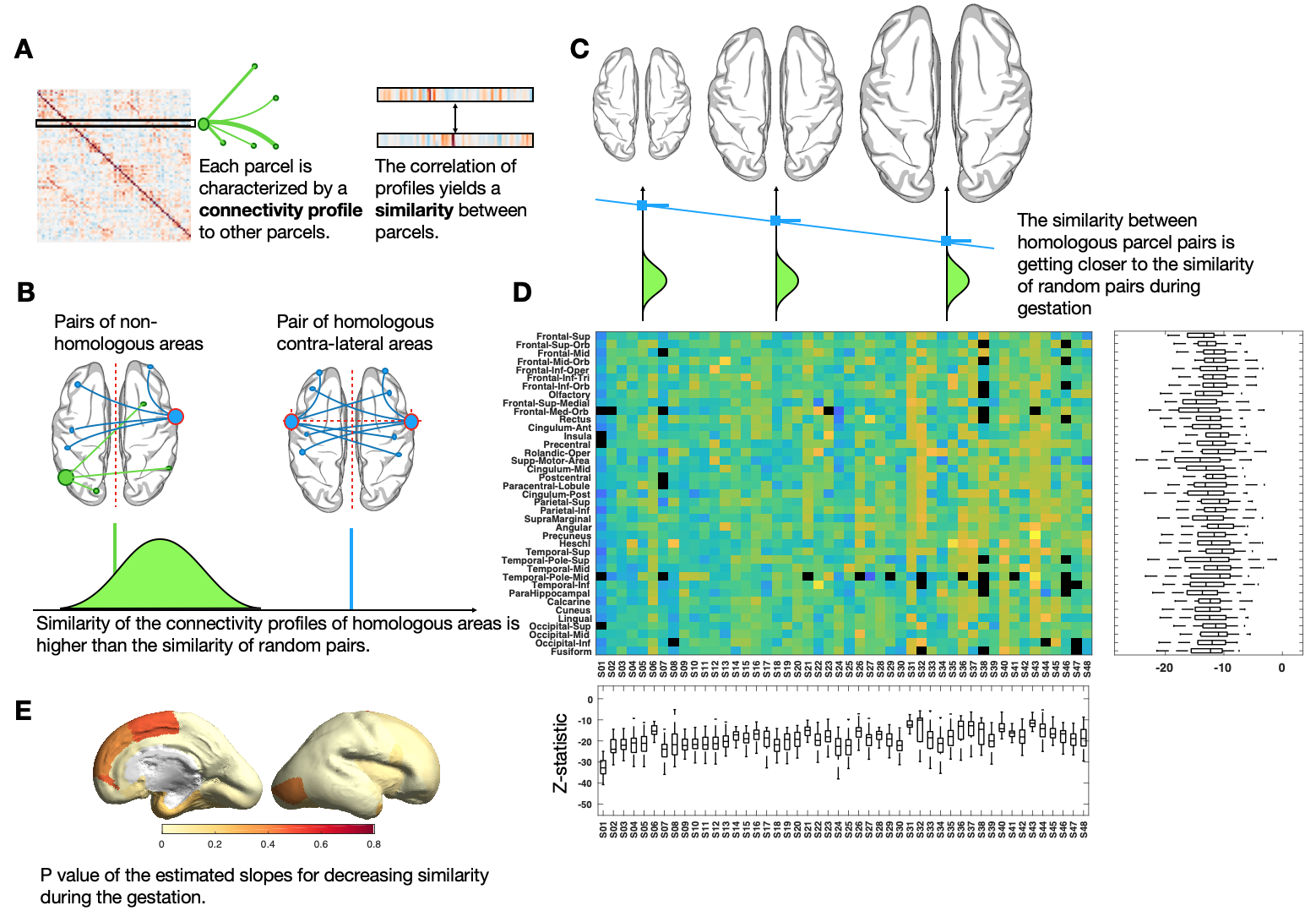

Supplement: SourceFiles_bhac446 [file sourcefiles_bhac446.zip › Figures/Similarity_ffmri_conn3.png]

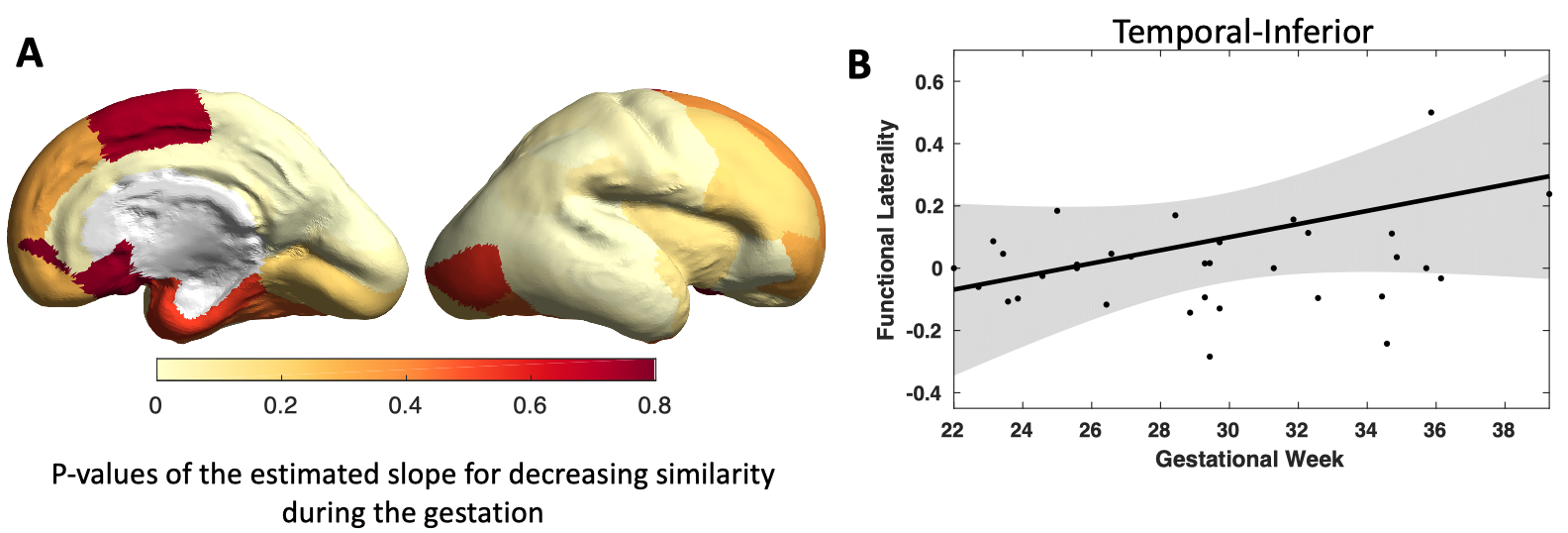

Supplement: SourceFiles_bhac446 [file sourcefiles_bhac446.zip › Figures/robustness.png]

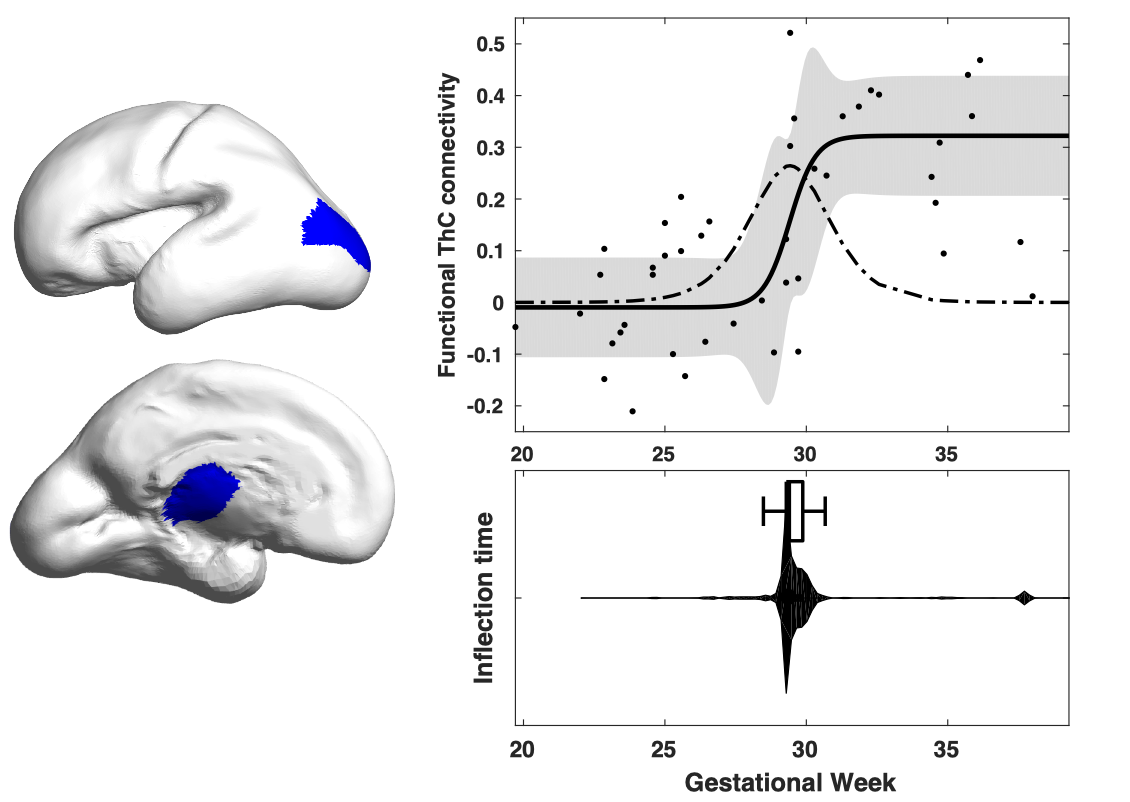

Supplement: SourceFiles_bhac446 [file sourcefiles_bhac446.zip › Figures/InflectionTime.png]
